# Adaptive robot climbing with magnetic feet in unknown slippery structure

**DOI:** 10.3389/frobt.2022.949460

**Published:** 2022-08-29

**Authors:** Jee-eun Lee, Tirthankar Bandyopadhyay, Luis Sentis

**Affiliations:** ^1^ Human Centered Robotics Lab, Department of Aerospace Engineering and Engineering Mechanics, The University of Texas at Austin, Austin, TX, United States; ^2^ The Robotics and Autonomous Systems Group, DATA61, CSIRO, Brisbane, QLD, Australia

**Keywords:** trajectory optimization, climbing robots, whole-body control, legged robots, optimization, parameterization, adaptation

## Abstract

Firm foot contact is the top priority of climbing robots to avoid catastrophic events, especially when working at height. This study proposes a robust planning and control framework for climbing robots that provides robustness to slippage in unknown environments. The framework includes 1) a center of mass (CoM) trajectory optimization under the estimated contact condition, 2) Kalman filter–like approach for uncertain environment parameter estimation and subsequent CoM trajectory re-planing, and 3) an online weight adaptation approach for whole-body control (WBC) framework that can adjust the ground reaction force (GRF) distribution in real time. Though the friction and adhesion characteristics are often assumed to be known, the presence of several factors that lead to a reduction in adhesion may cause critical problems for climbing robots. To address this issue safely and effectively, this study suggests estimating unknown contact parameters in real time and using the evaluated contact information to optimize climbing motion. Since slippage is a crucial behavior and requires instant recovery, the computation time for motion re-planning is also critical. The proposed CoM trajectory optimization algorithm achieved state-of-art fast computation *via* trajectory parameterization with several reasonable assumptions and linear algebra tricks. Last, an online weight adaptation approach is presented in the study to stabilize slippery motions within the WBC framework. This can help a robot to manage the slippage at the very last control step by redistributing the desired GRF. In order to verify the effectiveness of our method, we have tested our algorithm and provided benchmarks in simulation using a magnetic-legged climbing robot Manegto.

## 1 Introduction

Climbing robots can keep humans away from dangerous tasks such as inspection of vertical structures. Various types of climbing robots are developed with different adhesion modalities: suction Tummala et al. (2002), biologically inspired adhesion, for example, micro-splines Spenko et al. (2008); Parness et al. (2017) and micro-fibrillar Kim et al. (2007). Magnetic adhesion is predominant in industry Jose et al. (2018), for maintenance and inspection purposes of ferromagnetic surfaces. Robots with wheels Tavakoli et al. (2013); Eich and Vögele (2011) and continuous tracks Kermorgant (2018) are often used with magnetic adhesion but these robots have difficulties in traversing complex 3D structure. Legged platforms, on the other hand, offer greater mobility given their ability to execute discontinuous contact transition Bandyopadhyay et al. (2018). However, these benefits are achieved at the cost of increased complexity requiring great intelligence to control a robot over the unstructured ground.

### 1.1 What do we need to concern about for climbing behaviors?

Most current studies on climbing robots focused on their design and mechanism and only a few studies have been conducted on dynamic robot climbing behavior. Several studies propose to utilize biologically inspired templates to generate climbing motions Brown et al. (2018), Lynch et al. (2012), but their implementations are limited to certain behavior and do not consider any constraint regarding safe holdings that should be guaranteed for stable climbing. Motion planning and control have also been studied for free-climbing Bretl (2006); Miller and Rock (2008) and vertical wall climbing Lin et al. (2019); Lin et al. (2018). However, most works are also limited since the problems are formulated quasi-static to avoid computational complexity.

Apparently, the climbing and walking behavior of legged robots share a common mechanism: multi-contact transition to relocate their footholds to the proper position. It is more challenging for climbing robots as they need to hold onto an inclined surface while lifting their entire mass. Besides, given that most climbing robots are developed to inspect in an unknown environment, assumed adhesion and friction quality can often be different from the real values. Therefore, the need for a method to avoid falling and deal with unexpected slippage cannot be overemphasized especially for climbing robots.

### 1.2 How can we deal with slippage using robot control?

A combination of walking pattern parameter adaptation (long-term strategy) and force control (short-term strategy) to compensate for slippages is effective for robots walking on a slippery ground Takemura et al. (2005). However, predefined walking pattern adaptation can recover only from the limited amount of slippage. Instead, the idea to handle slip force as disturbance acting on a robot is suggested by Kaneko et al. (2005). A humanoid can avoid falling by estimating slip force *via* observer and compensating for the reactive motion caused by slip force. Focchi et al. (2018); Jenelten et al. (2019) proposed slip detection and recovery mechanism for quadruped robots developed to be employed in a WBC framework. However, both methods can still fail to recover from traction loss without proper centroidal motion re-planning to generate required GRFs to keep contact firm.

#### 1.2.1 Centroidal trajectory optimization

This study proposes to re-plan CoM motion *via* trajectory optimization to address the above limitations. Generating the proper CoM motion plays a critical role in vertical climbing Brown et al. (2018), though most studies achieve it *via* biologically inspired motion pattern. Trajectory optimization with centroidal dynamics is becoming popular in locomotion community Orin et al. (2013); Kim et al. (2019); Carpentier and Mansard (2018); Dai et al. (2014) due to its availability to handle friction condition simpler than solving full-body dynamics. Given the estimated adhesion force and friction coefficient, we can help a robot remain firm in contact by generating proper CoM motion. For example, by decelerating CoM in the gravity direction or normal to surface direction, we can relatively increase the normal contact force and reduce the tangential friction force at poor contact.

One well-known strategy to simplify centroidal dynamics optimization is the convexification of the system Ponton et al. (2018). By approximating non-convex quadratic constraint in a discrete-time system, it can obtain optimal CoM trajectory given multi-contact scenarios in 1 s for around 100 time steps. Another effective way to achieve fast computation is parameterization. Winkler et al. (2018); Ahn et al. (2021) leveraged phase-based parameterization to simplify trajectory description. Then, they formulate the problem to automatically determine the gait sequence, step timings, footholds, and swing-leg motions by solving nonlinear optimization in a discrete-time system. Though they provide the more generic form, the problem remains non-convex, and thus it takes about 4 s to be solved. Fernbach et al. (2020) utilized both parameterization and convexification *via* Bezier curves. However, since the whole trajectory over the multi-contact sequence is determined by one 3d vector decision variable, the generated motion could be limited. Also, the double description (DD) method is known to be unstable means to compute the linear constraints, which can be critical in robotics.

The proposed centroidal trajectory optimization leverages phase-based parameterization and several assumptions are applied to convexify the problem. Also, we showed that inequality constraints hold over the entire time horizon if it holds at the boundary of the function. Reduced dimension of constraints compared to that of the discrete-time domain provides faster computation. As a result, the proposed CoM trajectory optimization algorithm achieves a state-of-the-art fast computation simultaneously with an online contact parameter estimation algorithm to update the CoM trajectory immediately.

#### 1.2.2 QP-based whole-body control framework

Whole-body control (WBC) is a generic task-oriented control method that is particularly useful in controlling redundant robots like legged robots Khatib et al. (2004). A multilevel hierarchical control structure can be established *via* torque-based whole-body control, which allows humanoid robots to interact with the environment Sentis et al. (2010) compliantly. Optimization-based approaches have been popular for its ability to incorporate inequality constraints addressed within full-body dynamics Escande et al. (2014); Feng et al. (2015); Kim et al. (2020). Task hierarchy can be formulated as hierarchical least-square quadratic problems Escande et al. (2014); Bellicoso et al. (2016), but also can be considered *via* weighted cost terms in a single quadratic program Feng et al. (2015); Wiedebach et al. (2016). Even though weighted QP formulation can reduce the number of optimization problems to be solved, heuristically determined weights can violate the priority.

Whole-body locomotion controller (WBLC) leveraged a projection-based approach to consider equality task hierarchy and a QP formulation to find the command satisfying inequality constraints and full-body dynamics Kim et al. (2020). WBLC has successfully demonstrated dynamic locomotion of passive ankled robots. However, heuristic weights in QP formulation can violate the task hierarchy considered in task-priority projection-based inverse kinematics, especially when external disturbances or uncertainties exist in the environment. Lee et al. (2021) proposes a feedback gain adaptation method to stabilize external disturbances in WBLC based on the stability analysis. In this study, we suggest online weight adaption in QP formulation to address uncertainties, especially those that cause slippage. By regulating weights regarding reaction force depending on the slip level, we can enforce QP to find the command robust to the slippage.

### 1.3 Contribution

We summarize the contributions of this study as follows:

• We devise a state-of-the-art fast CoM trajectory optimization, which can be solved in 50 *μ*s for one-step climbing motion. By using phase-based parameterization and several assumptions to convexify the centroidal dynamics, the problem to determine CoM trajectory while satisfying the friction and adhesion condition during the swing phase can be solved about two orders of magnitude faster than other existing methods.
• We propose a Kalman filter–like approach for estimating unknown friction coefficient and (magnetic) adhesion force at slippery contact. Based on the contact force measurement, we linearize the observation model and formulate the system propagating itself to apply Kalman filter algorithm. This can be used in combination with our state-of-the-art fast algorithm to re-plan CoM trajectory to generate an instant slip recovery motion.
• We provide an online weight adaptation approach to be used in a QP-based WBC framework to stabilize the slippage instantly. We increase the normal contact force and decrease the tangential force by regulating the weights for ground reaction force with respect to the slippage rate.

Altogether, our main contribution is an integrated framework that provides planning and control strategies for robot climbing robust to the unknown environment. The proposed CoM trajectory optimization can be solved in real time allowing instant and effective slippage recovery. In combination with an online weight adaptation, the framework yields the best results in dealing with unknown slippery condition.

### 1.4 Organization of the article

This article is structured as follows:

• Problem definition (Section 2): This section introduces the robot climbing problem. An overview of our problem solving is also described in this section with the summary of WBLC as preliminary studies for the proposed framework.
• Multi-contact CoM trajectory generation for climbing (Section 3): This section describes the proposed CoM trajectory optimization method. Details are addressed from parameterization to approximations and applied linear algebra tricks step by step.
• Contact parameter estimation and CoM re-planning (Section 4): This section develops a slip reflex strategy when the slippage occurs. The overall procedure can be described as follows: 1) Friction coefficient and adhesion force in slippery contacts are estimated *via* either solving the least-square problem or the Kalman filter–like approach. 2) Given the re-evaluated friction coefficient and adhesion force, CoM re-planning is performed through the revised CoM trajectory optimization described in Section 3 to satisfy the new constraints.
• Online weight adaptation to stabilize slippery motions (Section 5): This section provides an online weight adaptation method that can be used in QP-based WBC. Defining the slippage rate that determines how the slip is severe, the weights can be adjusted to reduce slip behaviors.
• Experimental validation (Section 6): This section shows the performance of the proposed method *via* simulation experiment using a magnetic legged climbing robot Manegto. We have tested our algorithm and provided benchmarks. Several multi-contact scenarios including the different shapes of the climbing structure and various settings of the contact environment were used for the test.

## 2 Problem definition

In this study, we are aiming to solve a one-step climbing problem that can be described as follows: given the initial configuration of a robot in static equilibrium and given the next foot placement, the desired CoM position can be calculated—we chose the position close to the initial configuration and far from singularity—and we want to generate optimal trajectories for climbing and design the control laws robust to the environment uncertainties, especially the slip phenomenon.

### 2.1 Overview

**FIGURE 1:**
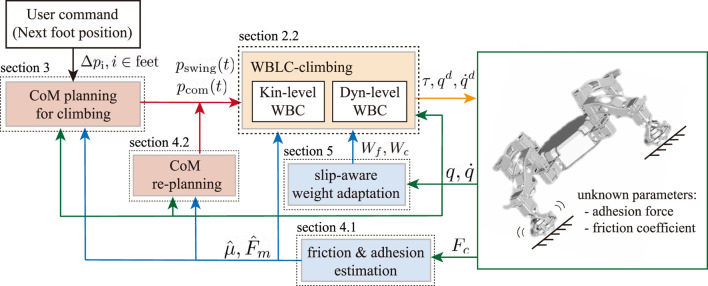
Control architecture.

Figure 1 shows an overview of the proposed control architecture. This framework is developed based on a whole-body locomotion controller (WBLC) Kim et al. (2020), which takes prioritized tasks as an input and outputs torque command with desired joint position and velocity commands at the instant time horizon. Usually, tasks prioritized in the following order are widely used in a legged robot: 1) maintaining rigid contact, 2) tracking CoM (centroidal) trajectory, 3) tracking swing foot trajectory, and 4) staying close to the initial configuration. Both CoM trajectory and swing foot trajectory are simply described as a straight line or parabola in most WBC works. However, since CoM trajectory can play an important role in generating required GRF in climbing motion, we propose to generate CoM motion *via* trajectory optimization. In addition, to deal with the slippage caused by the unknown environment, the estimation of adhesion force and friction coefficient is applied to the slipping contact. The re-evaluated parameters are updated in WBC that computes torque commands that satisfies friction constraints associated with the estimated friction coefficient and full-body dynamics affected by estimated magnetic adhesion. CoM re-planning could be also needed to generate the GRF that lies in the associated friction cone. Finally, a slip-aware online weight adaptation approach is integrated to the work with quadratic programming (QP) formulation in WBC to stabilize slippery motion instantly.

### 2.2 Whole-body locomotion controller extended to climbing

In this section, we first review WBLC with an extended formulation for climbing robots, especially those that use magnetism for adhesion, for example, Magneto Bandyopadhyay et al. (2018). WBLC proposed in Kim et al. (2020) solves the WBC problem by two sequential blocks: a *Kinematic-level WBC* that considers task hierarchy in inverse kinematics *via* projection-based method and a *Dynamic-level WBC* to compute torque command that satisfies all the dynamics constraints by solving quadratic programming (QP).

#### 2.2.1 Magnetic climbing dynamics

Magneto is developed to climb the wall using a switchable electromagnet attached to each foot. Each leg of Magneto consists of actuated joints for actuators and passive joints for gimbals. Given a configuration space $Q\in R^{n}$ and an input space $\;U\in R^{n_{a}}$ , the dynamics of a robot can be formulated as follows:

$$M\left(q\right)\ddot{q}+b\left(q,\dot{q}\right)=S_{a}\tau_{a}+J_{c}{\left(q\right)}^{⊺}F_{c}+J_{m}{\left(q\right)}^{⊺}F_{m},$$ (1)

where q∈Q , $M\left(q\right)\in S_{++}^{n}$ , $b\left(q,\dot{q}\right)\in R^{n}$ , and $\tau_{a}\in U$ denote the joint vector, mass/inertia matrix, sum of Coriolis/centrifugal force and gravitational forces, and actuator command, respectively. $S_{a}\in R^{n\times n_{a}}$ denotes the selection matrix indicating the index set of actuated joints, which maps ***τ***_*a*_ into the generalized forces. $F_{c}\in R^{6n_{c}}$ is a vertically concatenated contact wrench vector, where *n*_*c*_ is the number of contacts and the corresponding contact Jacobian is represented as $J_{c}\left(q\right)\in R^{6n_{c}\times n}$ . Similarly, magnetic force applied to the climbing robot for adhesion can be represented as a stacked wrench vector $F_{m}\in R^{6n_{m}}$ and the corresponding Jacobian matrix $J_{m}\left(q\right)\in R^{6n_{m}\times n}$ , where *n*_*m*_ is the number of activated magnetic mechanisms. In this study, we have assumed that both the contact forces and magnetic forces are applied at the center of each foot if and only if contact is made; thus, we have *n*_*c*_ = *n*_*m*_ and **J**_*c*_(**q**) = **J**_*m*_(**q**). Then the ground reaction force wrench can be represented as **F**_*r*_ = **F**_*c*_ + **F**_*m*_; the sum of forces are applied to the contacts.

#### 2.2.2 Kinematic level WBC

A kinematic level WBC computes the desired joint command $q^{d}\in R^{n}$ given the tasks defined in hierarchy. Let **J**_*k*_(**q**) and $x_{k}^{\text{des}}$ denote *k*th prioritized task Jacobian and the desired position. Then Δ**q**_*k*_, the change of joint configuration related to the *k*th task iteration, can be obtained by the propagation below:

$$\begin{array}{cc} J_{k|k-1}\left(q\right) & =J_{k}\left(q\right)N_{k-1}\left(q\right),\\ \Delta q_{k} & =J_{k|k-1}^{†}\left(x_{k}^{\text{des}}-x_{k}-J_{k}\left(q\right)\Delta q_{k-1}\right),\\ N_{k}\left(q\right) & =N_{k-1}\left(q\right)-J_{k|k-1}{\left(q\right)}^{†}J_{k|k-1}\left(q\right),\\ N_{0}\left(q\right) & =I_{n\times n}, \end{array}$$ (2)

where **N**_*k*_(**q**) and **J**_*k*|*k*−1_ represent *k*th task null-space projection and prioritized Jacobians. Then the desired joint position can be obtained by $q^{d}=q+\sum_{k=1}^{N}\Delta q_{k}$ in consideration of task priority. Similarly, ${\dot{q}}^{d}=\sum_{k=1}^{N}{\dot{q}}_{k}^{d}$ and ${\ddot{q}}^{d}=\sum_{k=1}^{N}{\ddot{q}}_{k}^{d}$ can be obtained from the following propagation:

$$\begin{array}{cc} {\dot{q}}_{0}^{d} & =0,\;{\ddot{q}}_{0}^{d}\;=\;0,\\ {\dot{q}}_{k}^{d} & =J_{k|k-1}^{†}\left({\dot{x}}_{k}^{d}-J_{k}\left(q\right){\dot{q}}_{k-1}^{d}\right),\\ {\ddot{q}}_{k}^{d} & =J_{k|k-1}^{†}\left({\ddot{x}}_{k}^{d}-{\dot{J}}_{k}\left(q,\dot{q}\right)\dot{q}-J_{k}\left(q\right){\ddot{q}}_{k-1}^{d}\right). \end{array}$$ (3)

#### 2.2.3 Dynamic level WBC

A dynamic level WBC calculates the desired torque command and modified joint command that satisfies dynamic constraints based on the optimization framework. This can be achieved by solving QP formulated as follows:

$$\begin{array}{c} \underset{\delta \ddot{q},F_{r},{\ddot{x}}_{c}}{\min},\;\delta {\ddot{q}}^{⊤}W_{\ddot{q}}\delta \ddot{q}+F_{r}^{⊤}W_{f}F_{r}+{\ddot{x}}_{c}^{⊤}W_{c}{\ddot{x}}_{c},\\ \text{subject to}\;M\left(q\right)\ddot{q}+b\left(q,\dot{q}\right)=S_{a}\tau_{a}+J_{c}{\left(q\right)}^{⊺}F_{c}+J_{m}{\left(q\right)}^{⊺}F_{m},\\ \ddot{q}={\ddot{q}}^{d}+K_{d}\left({\dot{q}}^{d}-\dot{q}\right)+K_{p}\left(q^{d}-q\right)+\delta \ddot{q}\\ \ddot{x_{c}}=J_{c}\ddot{q}+{\dot{J}}_{c}\dot{q},\\ F_{r}=F_{c}+F_{m},\\ U\left(\mu \right)F_{c}\geq 0,\\ \tau^{\text{min}}\leq \tau \leq \tau^{\text{max}}, \end{array}$$ (4)

where $W_{\ddot{q}}=\mathrm{diag}\left(w_{\ddot{q}}^{1},\ldots ,w_{\ddot{q}}^{n}\right)$ , $W_{f}=\mathrm{diag}\left(w_{f}^{1},\ldots ,w_{f}^{6n_{c}}\right)$ , and $W_{c}=\mathrm{diag}\left(w_{c}^{1},\ldots ,w_{c}^{6n_{c}}\right)$ are weight matrices corresponding to the joint acceleration relation $\delta \ddot{q}\in R^{n}$ , ground reaction force $F_{r}\in R^{6n_{c}}$ , and contact acceleration ${\ddot{x}}_{c}\in R^{6n_{c}}$ . $K_{p},K_{d}\in S_{+}^{n}$ are feedback gains corresponding to the desired joint commands $q^{d},{\dot{q}}^{d},{\ddot{q}}^{d}\in R^{n}$ obtained by Kinematic Level WBC, and $U\left(\mu \right)\in R^{17n_{c}\times 6n_{c}}$ represents diagonally stacked rectangular contact wrench cones (Caron et al., 2015) with respect to the contacts where the friction coefficients are $\mu ={\left[\mu_{1},\ldots ,\mu_{n_{c}}\right]}^{⊤}$ . Note that all the force wrenches and corresponding Jacobians in the formulation are expressed from the local body frame. Finally, torque command limits are considered given the minimum and maximum torques of the actuators ***τ*** ^min^, ***τ*** ^max^. By solving the aforementioned QP, we can finally compute the low-level joint torque command that satisfies all the dynamics constraints given the prioritized task.

#### 2.2.4 Phase-based state machine

Phase-based state machines are used together with WBLC to control a legged robot. We defined four state machines for one-step climbing control: full-support, pre-swing transition, swing, and post-swing transition. Each state machine represents a WBLC controller defined to consider different contact phases—e.g., different contact dimensions and weight parameters—so that the multi-contact climbing motion can be controlled by using the proper controller. The details can be found in Kim et al. (2020).

## 3 Multi-contact CoM trajectory generation for climbing

This planner can cooperate with the WBLC framework by providing the optimal CoM trajectory for climbing motion as the first prioritized task. Given the current configuration and the next desired contact location, we want to find a CoM trajectory for one step climbing motion, which satisfies constraints established under the estimated adhesion forces and friction coefficients.

### 3.1 Centroidal dynamics with magnetic force and friction cone constraints

**FIGURE 2:**
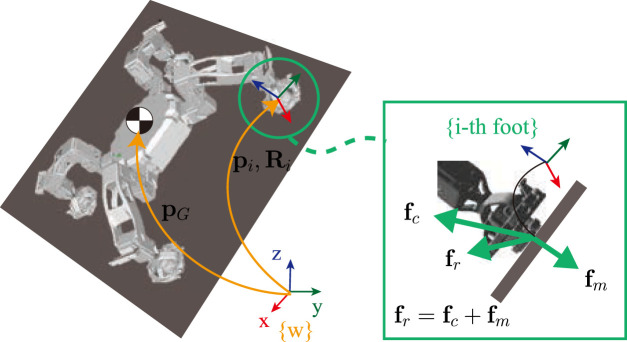
Free body diagram.

In this study, we formulated the problem with respect to the centroidal dynamics of a robot, which is commonly used in the legged robotics community as an effective way to simplify the robot dynamics while considering the contact constraints. By solving the Newton–Euler equations on the center of mass (CoM) of a robot, as described in free body diagram in Figure 2 we can formulate the centroidal dynamics as follows:

$$\begin{array}{ccc} \left[\begin{array}{c} mp_{G}\times \left({\ddot{p}}_{G}-g\right)+\dot{L}\\ m\left({\ddot{p}}_{G}-g\right) \end{array}\right] & = & \underset{i\in \text{Contact}}{\sum }\left[\begin{array}{c} p_{i}\times R_{i}f_{c,i}\\ R_{i}f_{c,i} \end{array}\right]+\underset{i\in \text{Magnet}}{\sum }\left[\begin{array}{c} p_{i}\times R_{i}f_{m,i}\\ R_{i}f_{m}^{i} \end{array}\right],\\ & = & \underset{R_{c}}{\underset{︸}{\overset{P_{c}}{\overset{︷}{\left[\begin{array}{c} p_{c_{1}}\times R_{c_{1}}\cdots \\ \;R_{c_{1}}\;\cdots \end{array}\right]}}}}\underset{f_{c}}{\underset{︸}{\left[\begin{array}{c} f_{c,c_{1}}\\ \vdots \end{array}\right]}}+\underset{R_{m}}{\underset{︸}{\overset{P_{m}}{\overset{︷}{\left[\begin{array}{c} p_{m_{1}}\times R_{m_{1}}\cdots \\ \;R_{m_{1}}\;\cdots \end{array}\right]}}}}\underset{f_{m}}{\underset{︸}{\left[\begin{array}{c} f_{m,m_{1}}\\ \vdots \end{array}\right]}}\\ & = & \left[\begin{array}{c} P_{c}\\ R_{c} \end{array}\right]f_{c}+\left[\begin{array}{c} P_{m}\\ R_{m} \end{array}\right]f_{m}, \end{array}$$ (5)

where *m* is the mass of the robot, **g** = [0,0,−9.81]^*⊤*^ is the gravity vector, $p_{G}\in R^{3}$ is the robot CoM position, and $\dot{L}\in R^{3}$ is angular momentum. $p_{i}\in R^{3}$ , $R_{i}\in \mathrm{SO}\left(3\right)$ are the position and orientation of the *i*th foot expressed in the world frame, which are used to map contact force $f_{c,i}\in R^{3}$ , and magnetic force $f_{m,i}\in R^{3}$ is expressed from the local foot frame. Finally, we defined $P_{c},R_{c}\in R^{3\times 3n_{c}},P_{m},R_{m}\in R^{3\times 3n_{m}}$ , $f_{c}\in R^{3n_{c}}$ , and $f_{m}\in R^{3n_{m}}$ to simply describe the equation using the stacked matrix and vector form.

In order to describe the friction that resists relative lateral motion between two solid surfaces in contact, the linearized Coulomb friction model was used in this study. It approximately provides a threshold value for friction force parallel to the surface as a function of the normal force. Based on the model, we can formulate the inequality constraints on contact forces as follows:

$$\begin{array}{c} Df_{c}\geq 0,\\ \text{where,}\;D=\mathrm{diag}\left(U_{f}\left(\mu_{c_{1}}\right),\cdots \;\right),\\ U_{f}\left(\mu \right)=\left(\begin{array}{ccccc} 0 & & 0 & & 1\\ 1 & & 0 & & \tilde{\mu }\\ -1 & & 0 & & \tilde{\mu }\\ 0 & & 1 & & \tilde{\mu }\\ 0 & & -1 & & \tilde{\mu } \end{array}\right)\text{ with }\tilde{\mu }=\frac{\mu }{\sqrt{2}} \end{array},$$ (6)

### 3.2 Phase-based CoM trajectory parameterization

**FIGURE 3:**
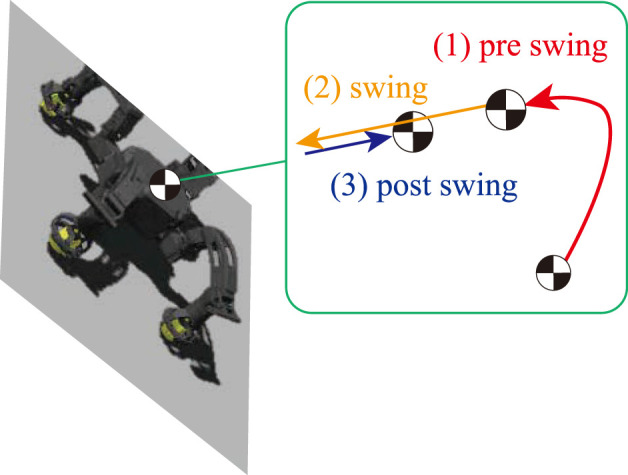
CoM trajectory represented by parameterized spline.

Legged motion is often represented as a sequence of contact phases. For instance, one step climbing motion for a quadruped can be described as the contact sequence of (1) full support/pre-swing (*n*_*c*_ = 4) → (2) swing (*n*_*c*_ = 3) → (3) full support/post-swing (*n*_*c*_ = 4). In this study, we composed CoM trajectories for climbing considering the contact sequence using cubic Hermite spline, where each piece represents the pre-swing, swing, and post-swing phase sequentially, as shown in Figure 3. Then CoM trajectory can be parameterized as three pieces of cubic Hermite polynomial defined by its duration(*T*), the initial and goal position of each node (**p**_*i*_, **p**_*g*_), and the velocities (**v**_*i*_, **v**_*g*_). This phase-based parameterization also matches with our WBLC (whole-body locomotion controller) framework well. Based on four state machines defined within WBLC (full support–transition–swing–transition), given that we can consider the transition as a part of the adjacent full support phase, the parameterized trajectory can be used according to the corresponding state machine.

However, we still need several assumptions to remove the non-convexity of the problem caused by the cross product term $p_{G}\times {\ddot{p}}_{G}$ . Del Prete et al. (2018) utilized the fact that the cross product of the parallel vectors is zero to solve the zero-step capturability problem. To be more specific, they assumed that the best strategy to stop CoM is to decelerate it in the direction CoM moves. Inspired by the strategy, we applied a similar assumption to our spline formulation. During the swing phase and post-swing phase, CoM moves along the straight line but accelerates in the opposite direction: ${\ddot{p}}_{G}^{\text{swing}}\left(t\right)=\alpha d,{\ddot{p}}_{G}^{\text{post swing}}\left(t\right)=\beta d$ , where *α*, *β* are constants with different signs and **d** is an unit vector indicating the direction of straight motion. Then given the initial and goal CoM position **p**_*a*_ and **p**_*b*_, respectively, CoM trajectory parameterization for one step climbing can be summarized as Table 1.

**TABLE 1:** Summary of phase-based CoM trajectory parameterization for one-step climbing motion. Given knots values described in the table, CoM trajectory can be computed *via* cubic Hermite polynomial (**p**_*HS*_(*t*; *T*, **p**_*i*_, **p**_*g*_, **v**_*i*_, **v**_*g*_)). Detailed equations are described in Appendix.

	(1) Pre swing	(2) Swing	(3) Post swing
*T* (duration)	*T*_1_	*T*_2_	*T*_3_
**p**_*i*_ (init position)	**p**_*a*_	$p_{1}=p_{b}+\left(\frac{1}{2}\beta T_{3}^{2}+\beta T_{3}T_{2}+\frac{1}{2}\alpha T_{2}^{2}\right)d$	**p**_2_
**p**_*g*_ (goal position)	**p**_1_	$p_{2}=p_{b}+\frac{1}{2}\beta T_{3}^{2}d$	**p**_*b*_
**v**_*i*_ (init velocity)	**0**	**v**_1_ = (−*βT*_3_ − *αT*_2_)**d**	**v**_2_
**v**_*g*_ (goal velocity)	**v**_1_	**v**_2_ = (−*βT*_3_)**d**	**0**

Note that vectors are denoted by boldface letters to avoid confusion with scalar variables. Given knots values described in the table.

### 3.3 Reformulation of centroidal dynamics based on spline parameterization

Now, we can reformulate the centroidal dynamics by substituting the parameterized CoM trajectory **p**_*HS*_(*t*; *T*, **p**_*i*_, **p**_*g*_, **v**_*i*_, **v**_*g*_), summarized in Table 1 into Eqs 5, 6. Given that slippage is most likely to happen during (2) swing and (3) post-swing phase, we assumed that parameterized CoM motion that satisfies friction conditions during (2) swing and (3) post-swing phase will also satisfy friction conditions during (1) pre-swing phase. Last, we assumed that both magnetic force **f**_*m*_ and contact force **f**_*c*_ are applied at the center of each foot frame for Magneto only if the foot is in contact, that is, $P_{c}=P_{m}$ and $R_{c}=R_{m}$ in Eq. 5. Then we have the following:

2) Swing:

$$\begin{array}{ccc} \left[\begin{array}{c} P_{s}-{\left[p_{b}\right]}_{\times }R_{s}\\ R_{s} \end{array}\right]f_{c} & = & \left[\begin{array}{c} \frac{1}{2}m{\left[g\right]}_{\times }{\left(T_{2}-t\right)}^{2}\\ mI_{3} \end{array}\right]\alpha d+\left[\begin{array}{c} \frac{1}{2}m{\left[g\right]}_{\times }T_{3}\left(T_{3}+2T_{2}-t\right)\\ 0 \end{array}\right]\beta d+\left[\begin{array}{c} \dot{L}-\left(P_{s}-{\left[p_{b}\right]}_{\times }R_{s}\right)f_{m}\\ -mg-R_{s}f_{m} \end{array}\right],\\ \Leftrightarrow \;A_{s}f_{c} & = & B_{sa}\left(t\right)\alpha d+B_{sb}\left(t\right)\beta d+c_{s},\;\forall t\in \left(0,T_{2}\right), \end{array}$$ (7)

$$D_{s}f_{c}\geq 0$$ (8)

where $P_{s},R_{s}\in R^{3\times 9}$ represent the configuration matrices( $P_{c},R_{c}$ in Eq. 5) mapping the force vectors $f_{c},f_{m}\in R^{9}$ during the swing phase. Finally, we denoted the coefficients of the obtained parameterized centroidal dynamics during the swing phase as $A_{s}\in R^{6\times 9}$ , $B_{sa}\left(t\right),B_{sb}\left(t\right)\in R^{6\times 3}$ , $c_{s}\in R^{6}$ , and the corresponding friction cone matrix as $D_{s}\in R^{15\times 9}$ .

3) Post swing:

$$\begin{array}{ccc} \left[\begin{array}{c} P_{f}-{\left[p_{b}\right]}_{\times }R_{f}\\ R_{f} \end{array}\right]f_{c} & = & \left[\begin{array}{c} \frac{1}{2}m{\left[g\right]}_{\times }{\left(T_{3}-t\right)}^{2}\\ mI_{3} \end{array}\right]\beta d+\left[\begin{array}{c} \dot{L}-\left(P_{f}-{\left[p_{b}\right]}_{\times }R_{f}\right)f_{m}\\ -mg-R_{f}f_{m} \end{array}\right],\\ \Leftrightarrow \;A_{f}f_{c} & = & B_{f}\left(t\right)\beta d+c_{f},\;\forall t\in \left(0,T_{3}\right), \end{array}$$ (9)

$$D_{f}f_{c}\geq 0,$$ (10)

where $P_{f},R_{f}\in R^{3\times 12}$ represent the configuration matrices mapping the force vectors $f_{c},f_{m}\in R^{12}$ during the post swing phase. $A_{f}\in R^{6\times 12}$ , $B_{f}\left(t\right)\in R^{6\times 3}$ , and $c_{f}\in R^{6}$ are coefficients of the parameterized centroidal dynamics during the post-swing phase and $D_{f}\in R^{20\times 12}$ is the corresponding friction cone matrix.

### 3.4 Problem solution for parameterized CoM trajectory generation

By applying parameterized CoM spline to the centroidal dynamics, the problem of determining CoM trajectory during one step climbing can be simplified as a problem to find *α*, *β*, **d** that satisfies Eqs 7–10. However, the problem is still non-linear since we have multiplication of variables, *α* **d**, *β* **d** in equation. Also, the different dimension of contact force vector (**f**_*c*_) in (7, 8) and (9, 10) makes a problem hard to be solved. Along with the assumptions we made in the CoM trajectory parameterization, we need several more tricks to solve the problem.

#### 3.4.1 Pre-determining the ratio of accelerations

First, we pre-determine $\gamma =\frac{\alpha }{\beta }$ , the ratio of the accelerations during the swing phase and the post swing phase. This way, we can linearize the problem by decoupling the constraint between *α* **d** and *β* **d**. We, specifically, chose *γ* that minimizes the maximum distance between the CoM goal position and the trajectory over the swing phase. This limits the trajectory to have minimal movement.

$$\begin{array}{ccc} \gamma^{*} & = & \arg\underset{\gamma }{\min}\left(\underset{t}{\max}|g_{\gamma }\left(t\right)|,\;t\in \left(0,T_{2}\right)\right),\\ where & & p_{s}\left(t\right)-p_{b}=g_{\gamma }\left(t\right)\cdot \beta d,\;t\in \left(0,T_{2}\right),\\ & & g_{\gamma }\left(t\right)=\frac{1}{2}\gamma {\left(T_{2}-t\right)}^{2}+T_{3}\left(T_{2}-t\right)+\frac{1}{2}T_{3}^{2}, \end{array}$$

Assuming *γ*∗ ≠ 0 and solving for *γ* < 0, we get the following:

$$\gamma^{*}=-\frac{T_{3}\left(T_{2}+T_{3}\right)+T_{3}\sqrt{{\left(T_{2}+T_{3}\right)}^{2}+T_{2}^{2}}}{T_{2}^{2}}.$$ (11)

#### 3.4.2 Contact force determination in centroidal dynamics

We took a simple solution for contact force in centroidal dynamics by mapping the weighted inverse of configuration matrix **A**_*s*_ and **A**_*f*_. In the centroidal dynamics described in Eqs. 7, 9, given the rank of the equation is six and **f**_*c*_ is either nine or 12, there is an infinite number of possible **f**_*c*_ tracking the given CoM trajectory. One effective way to handle a set of inequalities and equates is using the double description method as Fernbach et al. (2020) reformulate the problem. This way, we can find all feasible solutions based on the numerical conversion between the convex hull description method, but at the same time, it is known to be sometimes computationally unstable, which can be problematic in robotics. Instead, we simply assumed that contact force can be easily obtained by mapping the weighted inverse matrix $A^{-1}=W^{-1}A^{⊤}{\left({AW}^{-1}A^{⊤}\right)}^{-1}$ to the dynamics. This method is quite reliable, and we can configure it to find the contact force as close to the center of the friction cone as possible by designing the weight matrix *W* considering the shape of friction cone *D*, where we provided the details in Appendix. Then by denoting **x** = *β* **d** and substituting $f_{c}=A_{f}^{-1}\left(B_{f}\left(t\right)\beta d+c_{f}\right)$ and $f_{c}=A_{s}^{-1}\left(\left(B_{sa}\left(t\right)\gamma^{*}+B_{sb}\left(t\right)\right)\beta d+c_{s}\right)$ into each inequality equation, we have two linear matrix inequalities (LMI) for each phase.

$$D_{s}A_{s}^{-1}\left(B_{sa}\left(t\right)\gamma^{*}+B_{sb}\left(t\right)\right)x+D_{s}A_{s}^{-1}c_{s}\geq 0,\;\forall t\in \left(0,T_{2}\right),$$ (12)

$$D_{f}A_{f}^{-1}B_{f}\left(t\right)x+D_{f}A_{f}^{-1}c_{f}\geq 0,\;\forall t\in \left(0,T_{3}\right).$$ (13)

By splitting the inverse matrix $A_{f}^{-1},A_{s}^{-1}$ into left and right column matrices and expressing **B**_*f*_(t), **B**_*sa*_(*t*), and **B**_*sb*_(*t*) as a function of *t*, Eqs (12) and (13) can be rewritten as follows:

$$\begin{array}{l} \left(\frac{1}{2}m\underset{f_{s}\left(t\right)}{\underset{︸}{\left(\gamma^{*}{\left(T_{2}-t\right)}^{2}+T_{3}\left(T_{3}+2T_{2}-t\right)\right)}}{D_{s}A_{s}^{-1}}_{1;3}{\left[g\right]}_{\times }+m\gamma^{*}D_{s}{A_{s}^{-1}}_{4;6}\right)x+D_{s}A_{s}^{-1}c_{s}\geq 0,\\ \left(\frac{1}{2}m\underset{f_{f}\left(t\right)}{\underset{︸}{{\left(T_{3}-t\right)}^{2}}}D_{f}{A_{f}^{-1}}_{1;3}{\left[g\right]}_{\times }+mD_{f}{A_{f}^{-1}}_{4;6}\right)x+D_{f}A_{f}^{-1}c_{f}\geq 0. \end{array}$$

#### 3.4.3 Applying necessary and sufficient condition to satisfy inequality over the given time horizon

Proposition 1. Given an inequality $\left(f\left(t\right)B_{1}+B_{2}\right)x+c\geq 0$ defined over a bounded function *f*_min_ ≤ *f*(*t*) ≤ *f*_max_, *∀t* ∈ (0, *T*), if an inequality holds at the boundary values $\left(f_{\text{min}}B_{1}+B_{2}\right)x+c\geq 0$ and $\left(f_{\text{max}}B_{1}+B_{2}\right)x+c\geq 0$ for **x**, then $\left(f\left(t\right)B_{1}+B_{2}\right)x+c\geq 0,\forall t\in \left(0,T\right)$

The detailed proof of Proposition 1 is provided in Appendix. Based on Proposition 1, the problem to find **x** that satisfies inequalities along *t* can be reduced to the inequalities at the boundary of quadratic functions $f_{f}\left(t\right)={\left(T_{3}-t\right)}^{2},t\in \left(0,T_{3}\right)$ and $f_{s}\left(t\right)=\gamma^{*}{\left(T_{2}-t\right)}^{2}+T_{3}\left(T_{3}+2T_{2}-t\right),t\in \left(0,T_{2}\right)$ . Then by stacking inequalities for *f*_min_ and *f*_max_, we finally obtained the condition for **x** = *β* **d**:

$$\underset{D_{x}}{\underset{︸}{\left[\begin{array}{c} \frac{1}{2}mf_{s,min}D_{s}{A_{s}^{-1}}_{1;3}{\left[g\right]}_{\times }+m\gamma^{*}D_{s}{A_{s}^{-1}}_{4;6}\\ \frac{1}{2}mf_{s,max}D_{s}{A_{s}^{-1}}_{1;3}{\left[g\right]}_{\times }+m\gamma^{*}D_{s}{A_{s}^{-1}}_{4;6}\\ \frac{1}{2}mf_{f,min}D_{f}{A_{f}^{-1}}_{1;3}{\left[g\right]}_{\times }+mD_{f}{A_{f}^{-1}}_{4;6}\\ \frac{1}{2}mf_{f,max}D_{f}{A_{f}^{-1}}_{1;3}{\left[g\right]}_{\times }+mD_{f}{A_{f}^{-1}}_{4;6} \end{array}\right]}}x+\underset{d_{x}}{\underset{︸}{\left[\begin{array}{c} D_{s}A_{s}^{-1}c_{s}\\ D_{s}A_{s}^{-1}c_{s}\\ D_{f}A_{f}^{-1}c_{f}\\ D_{f}A_{f}^{-1}c_{f} \end{array}\right]}}\geq 0.$$ (14)

#### 3.4.4 Parameterized CoM trajectory optimization

Given the predefined sequence of contact for one step climbing motion, we parameterized the CoM trajectory with three polynomials corresponding to each contact phase and found the sufficient condition for the parameters that satisfy the centroidal dynamics and linearized friction cone inequalities as described in Inequality 14. Based on parameterization and all the assumptions and tricks we apply to simplify the problem, the problem to determine CoM trajectory can be reduced to the quadratic programming for **x** as follows:

$$\begin{array}{c} min\;\|x{\|}^{2},\\ \text{subject to }\;D_{x}x+d_{x}\geq 0, \end{array}$$ (15)

where $x=\beta d\in R^{3}$ , $D_{x}\in R^{70\times 3}$ , and $d_{x}\in R^{70}$ .

## 4 Contact parameter estimation and CoM re-planning

### 4.1 Friction and magnetic adhesion force estimation

If we have an F/T sensor on the feet so that we can measure the reaction force at each contact, we can estimate the friction and magnetic adhesion force. Once the slippage is detected, then the frictional force can be formulated at the sliding foot as follows:

$$f_{t}=\sqrt{f_{x}^{2}+f_{y}^{2}},$$ (16)

$${\hat{f}}_{t}=\hat{\mu }\left(f_{z}+{\hat{f}}_{m}\right),$$ (17)

where ${\left[f_{x},f_{y},f_{z}\right]}^{⊤}$ is the measured contact force represented in the local foot coordinate, $f_{t}\in R$ is the measured friction force which is equivalent to the tangential contact force, and ${\hat{f}}_{t}\in R$ is the estimated friction force computed based on the normal contact force $\left(f_{z}\in R\right)$ , the estimated friction coefficient $\hat{\mu }\in R$ , and magnetic adhesion force $\left({\hat{f}}_{m}\in R\right)$ . Let us define the parameter we want to estimate as $\theta ={\left[\hat{\mu },\hat{\mu }{\hat{f}}_{m}\right]}^{⊤}$ and assume that we can access the T-period of time sampling data for the estimation. Then we can estimate the parameter by solving the least-square problem below:

$$\theta^{*}=\arg\underset{\theta }{\min}\underset{t=1:T}{\sum }\|f_{t}\left(t\right)-\mu \left(f_{z}\left(t\right)+f_{m}\right){\|}^{2}.$$ 18)

By solving the first order necessary condition (FONC), we get

$$\left[\begin{array}{cc} \sum f_{z}\left(t\right) & \sum 1\\ \sum f_{z}{\left(t\right)}^{2} & \sum f_{z}\left(t\right) \end{array}\right]\left[\begin{array}{c} \hat{\mu }\\ \hat{\mu }{\hat{f}}_{m} \end{array}\right]=\left[\begin{array}{c} \sum f_{t}\left(t\right)\\ \sum f_{t}\left(t\right)f_{z}\left(t\right) \end{array}\right].$$ (19)

However, the least square estimation can be problematic if the contact is unstable. This can give inconsistent estimation value, which can be sometimes even unusable. In order to treat this issue, we propose the Kalman filter–like approach for estimating the friction coefficient and magnetic adhesion force. We first assumed that the system is propagating itself and has a T-period of time sampling ground reaction force data as observation. Also, we assumed that the measured normal force can be used as an observation model rather than observed data to simply linearize the model. This way, we can estimate the environment parameters more robust to the noise.

$$\begin{array}{c} \theta_{k+1}=\theta_{k}+w_{k},\;w_{k}∼N\left(0,Q_{k}\right),\\ z_{k}=H_{k}\theta_{k}+v_{k},\;v_{k}∼N\left(0,R_{k}\right),\\ where\;z_{k}=\left[\begin{array}{c} f_{t}\left(t_{k}-T+1\right)\\ \vdots \\ f_{t}\left(t_{k}\right) \end{array}\right],\;H_{k}=\left[\begin{array}{cc} f_{z}\left(t_{k}-T+1\right) & 1\\ \vdots & \vdots \\ f_{z}\left(t_{k}-T+1\right) & 1 \end{array}\right] \end{array}.$$ (20)

Though the formulated system is non-linear, given that the observation model **H**_*k*_ is not a constant but a function of the observed data, we just considered it as a time-varying observation model to apply the Kalman filter algorithm. Then we have the following:

$$\begin{array}{ll} K_{k} & =\left(P_{k-1}+Q_{k}\right)H_{k}^{⊤}{\left(H_{k}\left(P_{k-1}+Q_{k}\right)H_{k}^{⊤}+R_{k}\right)}^{-1}\\ P_{k} & =\left(I-K_{k}H_{k}\right)\left(P_{k-1}+Q_{k}\right),\\ {\hat{\theta }}_{k} & ={\hat{\theta }}_{k-1}+K_{k}\left(z_{k}-H_{k}{\hat{\theta }}_{k-1}\right). \end{array}$$ (21)

### 4.2 CoM re-planning for slip reflex

When a robot climbs in an unknown environment, we can expect a robot to experience slip behavior when the uncertainty for a contact is high, for example, when the foot in contact was a swing foot in the previous step. If a slip is detected and the estimated friction coefficient and adhesion forces are significantly inferior, then we will need to re-plan the CoM trajectory for generating the desirable contact forces to keep rigid contact. The slip is supposed to be detected during the pre-swing or swing phase when the uncertainty for a contact is high. If slip is detected during the pre-swing phase, then the CoM trajectory optimization for re-planning will have the same formulation as the one solved in Section 3. The re-computed *α*, *β*, **d** for re-evaluated $\hat{\mu },{\hat{F}}_{m}$ can be used to update CoM trajectory. The only difference is the initial position and velocity of the CoM set to zero in the previous problem will be substituted into the current position and velocity of CoM.

#### 4.2.1 Revised CoM trajectory optimization algorithm for re-planning in the swing phase

The CoM re-planing problem can be defined as follows: given the current and the desired configuration; given the current and desired CoM position **p**_*a*_, **p**_*b*_; and given the current and desired contacts configuration $P_{s},P_{f},R_{s},R_{f}$ , find the CoM trajectory that satisfies the new friction condition. Let *t*_now_ be the time past since a robot started the swing phase, then we can re-parameterize the CoM trajectory for the remaining climbing motion as Table 2.

**TABLE 2:** Revised CoM parameterization for the swing phase re-planning.

	(2) Swing	(3) Post swing
*T* (duration)	$T_{2}^{\prime }=T_{2}-t_{\text{now}}$	*T*_3_
**p**_*i*_ (init position)	**p**_*a*_	**p**_1_
**p**_*g*_ (goal position)	$p_{1}=p_{a}+\frac{1}{2}\alpha T_{2}^{\prime 2}d$	**p**_*b*_
**v**_*i*_ (init velocity)	**0**	**v**_1_
**v**_*g*_ (goal velocity)	$v_{1}=\alpha T_{2}^{\prime }d$	**0**

Then the centroidal dynamics during the swing and post-swing phase can be rewritten as follows:

2) Swing:

$$\left[\begin{array}{c} P_{s}-{\left[p_{a}\right]}_{\times }R_{s}\\ R_{s} \end{array}\right]f_{c}=\left[\begin{array}{c} \left({\left[p_{a}\right]}_{\times }R_{s}-P_{s}\right)f_{m}+\dot{L}\\ -mg-R_{s}f_{m} \end{array}\right]+m\left[\begin{array}{c} \frac{1}{2}t^{2}{\left[g\right]}_{\times }\\ I \end{array}\right]\alpha d.$$ (22)

3) Post swing:

$$\begin{array}{ll} \left[\begin{array}{c} P_{f}-{\left[p_{1}\right]}_{\times }R_{f}\\ R_{f} \end{array}\right]f_{c} & =\left[\begin{array}{c} \left({\left[p_{a}\right]}_{\times }R_{f}-P_{f}\right)f_{m}+\dot{L}\\ -mg-R_{f}f_{m} \end{array}\right]+m\left[\begin{array}{c} f_{1}\left(t\right){\left[g\right]}_{\times }\\ f_{2}\left(t\right)I \end{array}\right]\alpha d,\\ where\;f_{1}\left(t\right) & =-\frac{1}{2}T_{2}^{\prime 2}\left(-2{\left(\frac{t}{T_{3}}\right)}^{3}+3{\left(\frac{t}{T_{3}}\right)}^{2}\right)+T_{2}^{\prime }\cdot T_{3}\left({\left(\frac{t}{T_{3}}\right)}^{3}-2{\left(\frac{t}{T_{3}}\right)}^{2}+\left(\frac{t}{T_{3}}\right)\right),\\ f_{2}\left(t\right) & =-\frac{1}{2}{\left(\frac{T_{2}^{\prime }}{T_{3}}\right)}^{2}\left(-12\frac{t}{T_{3}}+6\right)+\frac{T_{2}^{\prime }}{T_{3}}\left(6\frac{t}{T_{3}}-4\right). \end{array}$$ (23)

Then similar to the problem solving we described in Eq. 12 ∼(15), we can reformulate the optimization problem for **x** = *α* **d** with $D_{x}^{\prime }$ and $d_{x}^{\prime }$ defined similar to **D**_*x*_ and **d**_*x*_ in Eq. 14 as follows:

$$\begin{array}{c} min\;\|x{\|}^{2},\\ \text{subject to }\;D_{x}^{\prime }x+d_{x}^{\prime }\geq 0 \end{array}.$$ (24)

## 5 Online weight adaptation to stabilize slippery motions

In this section, we present an online weight adaptation approach to stabilize slippery motions by redistributing the contact force at each contact instantly. As discussed in the previous section, the final torque commands are computed by solving QP-based formulation in *Dynamic level WBC* block. Weights in QP formulation.

### 5.1 Slippage rate for weight adaptation

In order to update weight gains according to the slippage rate in contact, we need a way to measure the rate of slippage. In this study, we defined the slippage rate based on slip velocity at each contact as follows:

$$\begin{array}{ccc} \alpha_{s} & = & \left\{\begin{array}{cc} 1\; & \text{if }\|v_{\text{contact foot}}\|\leq v_{\text{threshold}}\\ \frac{\left\|v_{\text{contact foot}}\right\|}{v_{\text{threshold}}}\; & otherwise \end{array}\right.\\ v_{\text{contact foot}} & = & \text{LOW PASS FILTER}\left(J_{c}\left(q\right)\dot{q}\right). \end{array}$$

By defining the rate with the threshold, we can provide weight adaptation only if the slippage is large enough and the rate parameter itself is continuous as well.

### 5.2 Weight adaptation to the slippage

Based on the structure of WBLC, we formulated an extension version of WBLC for climbing in Section 2.2. Recalling that the cost function is designed to minimize the ground reaction force and tracking error:

$$\underset{\delta \ddot{q},F_{r},{\ddot{x}}_{c}}{\min},\;\delta {\ddot{q}}^{⊤}W_{\ddot{q}}\delta \ddot{q}+F_{r}^{⊤}W_{f}F_{r}+{\ddot{x}}_{c}^{⊤}W_{c}{\ddot{x}}_{c},$$

Let us say we have contact foot 1, 2, … , *c*. Then the corresponding slippage ratio and weight matrix can be described as follows:

$$\begin{array}{ccc} \alpha_{s} & = & \left({\alpha_{s}}_{1},\ldots ,{\alpha_{s}}_{c}\right),\\ W_{f} & = & \mathrm{diag}\left(w_{fx_{1}},w_{fy_{1}},w_{fz_{1}},\ldots ,w_{fx_{c}},w_{fy_{c}},w_{fz_{c}}\right). \end{array}$$

Typically, we used predefined weight parameters in WBC and usually took $w_{fx_{i}},w_{fy_{i}}=w_{xy}$ and $w_{fz_{i}}=w_{z}$ . Once we have foot in slippery contact, ${\alpha_{s}}_{i}>1$ , we can update the corresponding weight parameters based on the ratio online as follows:

$$w_{fx_{i}},w_{fy_{i}}=\left\{\begin{array}{c} {\alpha_{s}}_{i}w_{xy},\;\\ \frac{1}{{\alpha_{s}}_{i}}w_{xy},\; \end{array}\right.\text{and },w_{fz_{i}}=\left\{\begin{array}{cc} \frac{1}{{\alpha_{s}}_{i}}w_{z},\; & \text{if}\;{\alpha_{s}}_{i}>1,\\ {\alpha_{s}}_{i}w_{z},\; & \text{otherwise}. \end{array}\right.$$ (25)

## 6 Experimental validation

In this section, we focus on showing the performance of the proposed framework. In Section 6.1, we present how the proposed algorithm yields competitive performance against other CoM trajectory generation methods. Then, in Section 6.2, parameter estimation to identify unknown slippery condition is presented. Finally, in Section 6.4, we have demonstrated robot climbing behavior in unknown slippery conditions with and without the proposed strategies to evaluate the performance of the algorithms.

### 6.1 Comparison benchmarks of proposed CoM trajectory optimization

In order to show the performance of our CoM trajectory optimization, we choose to compare the computation time of the proposed algorithm with a state-of-the-art convexified formulation Ponton et al. (2018) and parameterized formulation (CROC) Fernbach et al. (2020). Generated CoM trajectory and corresponding ground reaction force distribution are also analyzed for comparison. The codes used in our benchmark modified for climbing is provided for CROC^1^ and convexified centroidal optimization^2^, and for the proposed method^3^.

For these benchmarks, we have performed a climbing simulation in two different structures.

**FIGURE 4:**
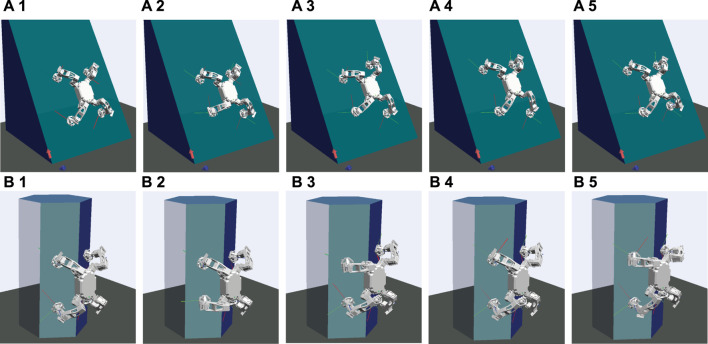
Multi-contact scenarios on **(A)** a flat slope at *α* =1rad and **(B)** a hexagonal structure. A robot is climbing up on each structure by moving its feet in the order of left bottom (1→2), right top (2→3), left top (3→4), and right bottom (4→5).

• The first structure described in Figure 4A is a flat slope inclined at *α* = 1 rad, where all the contacts of a robot are always on the same planar. One of the contacts is set particularly poor, where the environment parameter of the left bottom foot is set to *μ*_*s*_ = 0.3 and *f*_*m*_ = 30(N), while other feet are set to *μ*_*s*_ = 0.5 and *f*_*m*_ = 70(N).
• The second structure described in Figure 4B is a hexagonal structure, which leads a robot to climb with a set of non-co-planar contacts. All the parameters that determine the contact quality are given the same for all feet as *μ*_*s*_ = 0.5 and *f*_*m*_ = 70(N).

In each structure, a robot is climbing up on a wall by moving its feet in the order of left bottom, right top, left top, and right bottom foot. All the benchmark simulations were run on Ubuntu 18.04.5 LTS with Processor Intel® Core^TM^ i7-8700K CPU @ 3.70GHz × 12, Memory 16 GB. QuadProg++ (Goldfarb and Idnani (1983)) were used to solve the QP problem in our software.

As described in Table 3, the computation time of the proposed algorithm is about 4 ∼5 orders of magnitude faster than the convexified centroidal dynamics optimization method and two orders of magnitude faster than CROC. This result is natural considering that the dimension of convexified centroidal dynamics optimization is much larger because it solves the problem for the time discretization variables. On the other hand, parameterization can reduce the dimension of the problem effectively. Though CROC also uses parameterization, the double description (DD) method to handle inequality conditions in CROC takes a relatively long time. Instead, we utilized the weighted inverse matrices to determine contact forces in the centroidal dynamics equation, which helps us to find a reasonable solution two orders of magnitude faster. Furthermore, as the DD method is known to suffer from computational instability, which can cause a critical problem in robotics, the proposed solving method can be beneficial in terms of computation stability as well.

**TABLE 3:** Computation time comparison benchmark of CoM trajectory optimization for two different climbing scenarios between the proposed algorithm and other existing methods. Computation time is averaged over 100 tries for each scenario.

Computation time [ms] for flat slope climbing scenario(motion horizon = 0.65 s)					
	Swing foot	Left bottom	Right top	Left top	Right bottom
	Proposed method	5.22e-02	5.23e-02	5.45e-02	5.67e-02
Method	Ponton et al. (2018) (Δ*T* = 10ms)	2,400	2,230	2,280	2,330
	Ponton et al. (2018) (Δ*T* = 50ms)	313	295	303	314
	Fernbach et al. (2020)	7.51	7.32	7.81	7.35

Computation time [ms] for hexagonal structure climbing scenario(motion horizon = 0.8s)					
	Swing foot	Left bottom	Right top	Left top	Right bottom
	Proposed method	5.29e-02	5.78e-02	5.33e-02	5.91e-02
Method	Ponton et al. (2018) (Δ*T* = 50ms)	365	332	326	379
	Fernbach et al. (2020)	1.40	1.58	1.40	1.59

**FIGURE 5:**
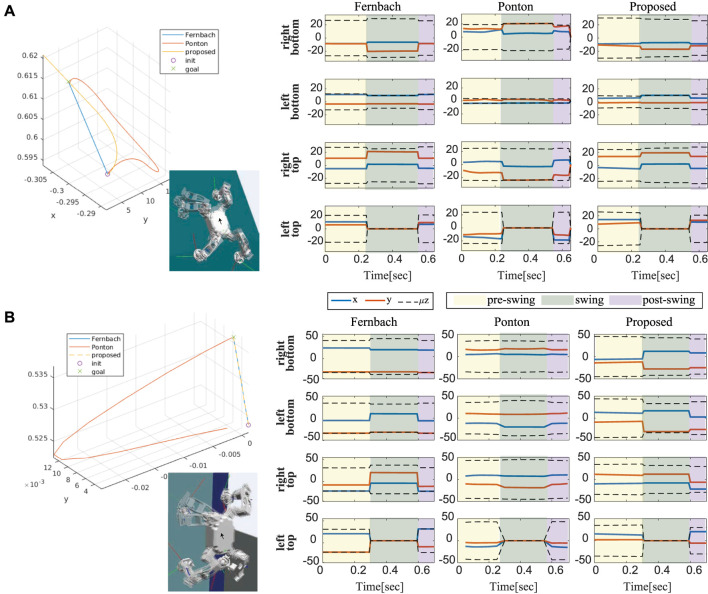
The comparison of centroidal climbing motion obtained by different algorithms. One-step climbing motions for the right top foot are compared on two structures [**(A)** a flat slope at α = 1 rad and **(B)** a hexagonal structure]. The plots on the right show the corresponding desired GRF distribution on each foot with friction inequality constraints.

Not surprisingly, the trajectory obtained by different algorithms was different as shown in Figure 5. It is natural, considering that there are always an infinite number of trajectories that satisfy constraints in many trajectory optimization problems. One of the advantages we can take from parameterization is that we can expect a shape of the trajectory planning. This is important in robotics since unexpected plans can often cause a critical problem. In addition, the more variables the problem has, the more weight parameters we need to tune. In Ponton et al. (2018), the obtained trajectories were sensitive to the choice of weights, resulting in another difficulty in weight tuning. Finally, as we obtain the contact force placed as close as possible to the center of the friction cone, we can see the desired contact forces obtained by the proposed method tend to have more margin in inequalities.

### 6.2 Parameter estimation

**FIGURE 6:**
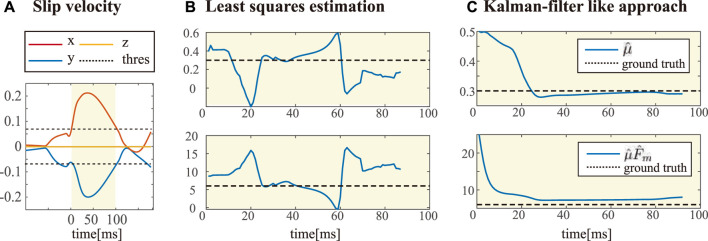
Estimation of unknown environment parameters $={\left[\mu ,\mu F_{m}\right]}^{⊤}$ during the slip condition. **(A)** shows the condition of the estimation activation. Once the contact velocity exceeds the threshold during the certain amount of time, we declare the slip detection so that the estimation can be executed. Yellow areas in the plots represent where the estimation is activated. **(B)** and **(C)** present the resulting estimation obtained by two different approaches, least-squares, and Kalman filter–like approach.

As described in Section 4, unknown environment parameters *μ* and *F*_*m*_ can be estimated based on the friction force model when a slip occurs. Figure 6 shows the parameter estimation based on two different algorithms. Once the contact velocity exceeds the threshold, we prepare the parameter estimation, assuming that there is a slip. Though we assumed that we can detect the slippage by thresholding the contact velocity computed based on the floating base configuration, this cannot be achieved in the real legged robot system. Instead, either comparing the velocity of the contact and the median of the velocity of all contacts Focchi et al. (2018) or a probabilistic state machine Jenelten et al. (2019) can be considered for slip detection in real robots.

Once the slip detection is triggered, we store the contact force data during 15 time samples. Then, we can estimate the unknown parameters $\theta ={\left[\mu ,\mu F_{m}\right]}^{⊤}$ that tell how slippery the surface is *via* either least-square estimation or Kalman filter-like approach. As you can see from Figure 6B, parameter estimation through the least-square estimation was not desirable; The observation of contact forces is too noisy even in simulation, and observation data captured during the traction loss doesn’t match with the friction force model we used for estimation. On the other hand, Kalman filter–like approach worked well with a small value for model covariance **Q**_*k*_ = diag([0.000004, 0.01]), and a relatively large value for observation covariance **R**_*k*_ = 0.25. From the initial guess of parameters, you can see it converges in 30ms to the ground-truth value in Figure 6C.

### 6.3 Slip-aware online weight adaption for QP based WBC

**FIGURE 7:**
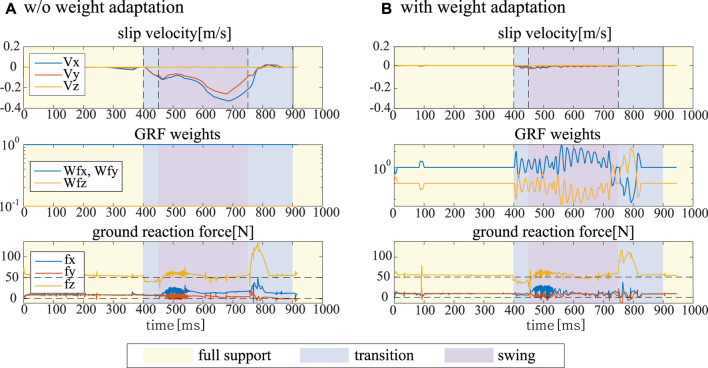
Slip phenomenon with and without an online weight adaptation. **(A)** shows the linear velocity and ground reaction force at the contact in slippery condition without any adaptation and **(B)** shows reduced slippage at the contact in the same condition with the proposed weight adaptation.

In Section 5, we present an online weight adaptation for QP-based WBC framework. Figure 7 shows a part of motion suffering from sliding. It describes how the proposed algorithm shapes the corresponding weights based on the slip velocity and how it affects the corresponding ground reaction forces. Once the slip velocity exceeds the threshold, it starts to change the weights, and this can help a robot to slightly redistribute contact forces to stop sliding.

### 6.4 Climbing in unknown slippery condition

In Section 4 and Section 5, we proposed CoM trajectory re-planning based on re-evaluated parameters and an online weight adaptation to stabilize a slip. Lastly, in this section, to verify the performance of the proposed control framework in terms of robustness to the unknown slippage, we examine the slip velocity of the foot in unknown slippery conditions with respect to the following 4 controllers:

• WBLC without any adaptation,
• WBLC with parameter estimation and CoM re-planning,
• WBLC with an online weight adaptation,
• WBLC with both methods.

In this simulation, the controller assumes the environment parameters for all feet to be *μ* = 0.5, *F*_*m*_ = 50*N*, while the parameters of the left bottom foot are set to *μ* = 0.3, *F*_*m*_ = 20*N*. The climbing scenario on a flat slope described in Figure 4A was used for this verification test.

**FIGURE 8:**
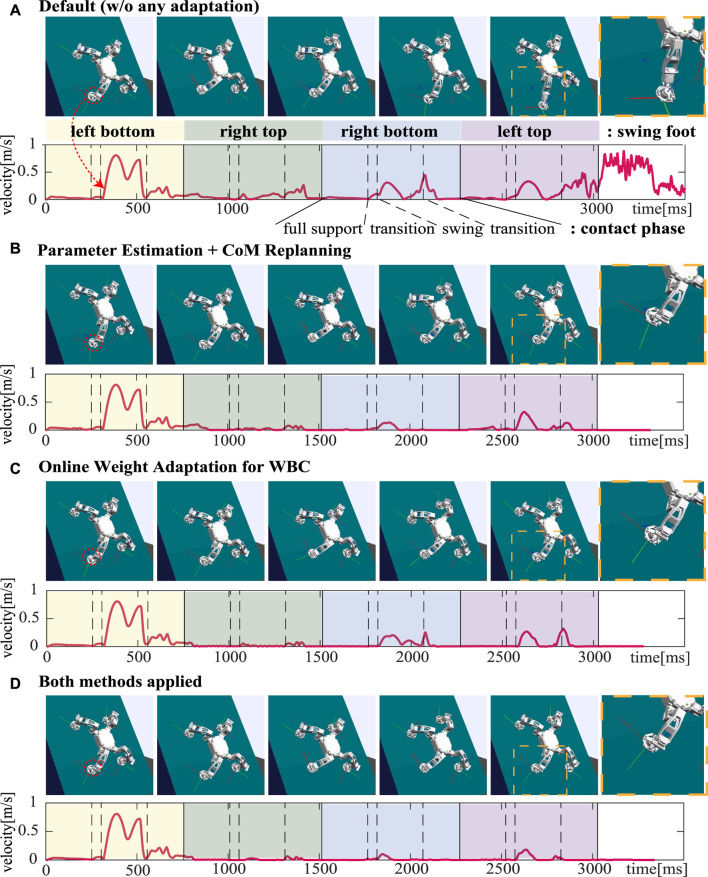
Comparative analysis on the slippage of the foot in unknown slippery conditions for each controller: **(A)** WBLC without any adaptation, **(B)** WBLC with unknown parameter estimation and CoM replanning (described in Section 4), **(C)** WBLC with online weight adaptation (described in Section 5), and **(D)** WBLC with both methods [applied in **(B)** and **(C)**]. Slip velocities of the left bottom foot and snapshots of the simulation are represented. After the first climbing step where the left bottom foot is used for swing, the velocity is supposed to be zero. Blue dots in the last zoom-in shots represent the desired foot position.

In Figure 8, the slip velocities of the left bottom foot for each controller are shown with snapshots of the simulation. As shown in Figure 8A, without any adaptation, the controller cannot compute the adequate trajectory and control satisfying the real friction constraints, and that eventually results in contact loss at the left bottom foot after four climbing steps are made. You can also see that a slip tends to occur during the swing phase and post swing transition where one of the feet is used for making a step.

**FIGURE 9:**
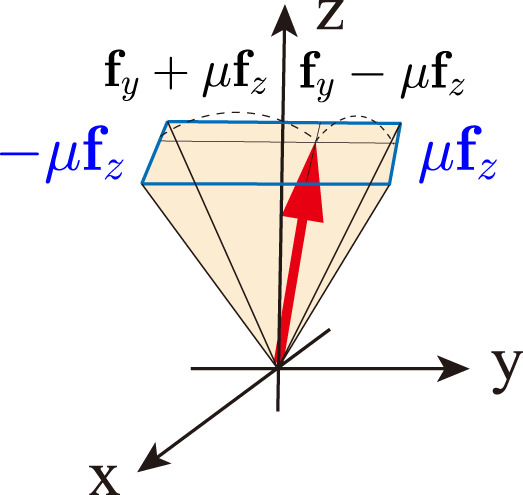
Linearized friction cone.

Figures 8B,C shows both CoM replanning and an online weight adaptation for WBC are effective to stabilize the slippery motion. The difference shown in the results for the two methods is that the online weight adaptation method faces the second slip once the slippery is stabilized especially around the start of the post-swing transition phase for each climbing step. That could be caused since the weights are initialized in every phase in our controller. Finally, as shown in Figure 8D, we validated that the slip can be prevented the most when both methods are applied.

## 7 Conclusion

In this study, we have presented several strategies to stabilize known and unknown slippery motion within a WBLC framework. In an effort to stabilize unknown slip, CoM re-planning based on unknown parameter estimation and online weight adaptation for WBC were presented. When the environment parameters are unknown and different from what we assume when we solve the problem, the obtained solution can suffer from unknown behaviors, for example, slip, traction loss, etc. Based on the friction force model, the friction coefficient and the friction force limit calculated from the adhesion force can be estimated when the contact is under the slip condition. Then, CoM re-planning is performed based on the proposed CoM trajectory optimization algorithm. The proposed CoM trajectory optimization achieved fast computation by leveraging a phase-based parameterization and we verified it by providing comparison benchmarks with other state-of-art algorithms. Therefore, the proposed CoM trajectory generation method allows for CoM re-planning in real time, and this can reduce the slip by generating adequate contact forces to increase the normal force and decrease the tangential force at slippery contact. The proposed strategies are shown to be effective to reduce slip through the simulation experiment.

Although we have shown that the proposed methods are fast and effective to stabilize the slippery motion, traction loss could be another issue that can cause devastating result especially for overhang climbing as you can see from the provided video. The proposed CoM optimization algorithm is expected to be extended to prevent a robot from those fetal movements. While working on this study, we also found that the wrong estimated parameter can make robot climbing more problematic, even though the estimation are sorely dependent on the noisy and uncertain observation. In addition, this study does not provide thorough theoretical analysis on the stability of the proposed method. In that in mind, instability resulting from the parameter estimation and stability analysis on weight adaptation should be addressed in our future research.

Lastly, our approach has not been tested yet on a real robot, although there could be a lot more challenges arising from hardware experiment. For example, in a real robot setup, a robot can always be susceptible to kinematic singularities, discretization of dynamics and model error, etc. Though we didn’t mention it in this study, we consider the singularity when we set the goal CoM position. We solve the optimization problem that is formulated to find the goal configuration far from singularities given the next foot placement, and we are using the goal CoM position obtained from the configuration. As for the dynamics errors, augmented PD control can be used along with the WBC to compensate for errors from the dynamics model. In addition, for legged robot system, it is hard to estimate the base configuration accurately as simulation, though we assumed that we can detect slip based on the floating based robot kinematics. However, we can use other measurement such as F/T sensor values or IMU values to define slippage ratio and apply it for online weight adaptation. Overall, thus verifying the feasibility to implement this work in real hardware setup is the next coming step, as well.

## Data Availability

The datasets presented in this study can be found in online repositories. The names of the repository/repositories and accession number(s) can be found at: https://github.com/jeeeunlee/ros-pnc/tree/devel-mpc-slip.

## Footnotes

1
https://github.com/jeeeunlee/ccroc_magneto

2
https://github.com/jeeeunlee/centroidal_sandbox_magneto

3
https://github.com/jeeeunlee/ros_pnc branch:mpc-devel-slip

## 8 Appendix

### 8.1 Cubic hermite spline

$$\begin{array}{ccc} p_{HS}\left(t;T,p_{i},p_{g},v_{i},v_{g}\right) & = & p_{i}\left(2{\left(\frac{t}{T}\right)}^{3}-3{\left(\frac{t}{T}\right)}^{2}+1\right)+v_{i}T\left({\left(\frac{t}{T}\right)}^{3}-2{\left(\frac{t}{T}\right)}^{2}+\frac{t}{T}\right)\\ & & +p_{g}\left(-2{\left(\frac{t}{T}\right)}^{3}+3{\left(\frac{t}{T}\right)}^{2}\right)+v_{g}T\left({\left(\frac{t}{T}\right)}^{3}-{\left(\frac{t}{T}\right)}^{2}\right),\\ v_{HS}\left(t;T,p_{i},p_{g},v_{i},v_{g}\right) & = & \frac{p_{i}}{T}\left(6{\left(\frac{t}{T}\right)}^{2}-6\frac{t}{T}\right)+v_{i}\left(3{\left(\frac{t}{T}\right)}^{2}-4\frac{t}{T}+1\right)+\frac{p_{g}}{T}\left(-6{\left(\frac{t}{T}\right)}^{2}+6\frac{t}{T}\right)+v_{g}\left(3{\left(\frac{t}{T}\right)}^{2}-2\frac{t}{T}\right),\\ a_{HS}\left(t;T,p_{i},p_{g},v_{i},v_{g}\right) & = & \frac{p_{i}}{T^{2}}\left(12\frac{t}{T}-6\right)+\frac{v_{i}}{T}\left(6\frac{t}{T}-4\right)+\frac{p_{g}}{T^{2}}\left(-12\frac{t}{T}+6\right)+\frac{v_{g}}{T}\left(6\frac{t}{T}-2\right). \end{array}$$

### 8.2 Weight matrix design for friction cone

Given the centroidal dynamics, the problem to find contact force that satisfies all the constraints can be formulated as follows:

$$\begin{array}{ccc} Find\;f_{c}\;\text{s.t. } & & Af_{c}=b,\\ & & Df_{c}\geq 0\;\text{where}\;D=\mathrm{diag}\left(U_{f}\left(\mu^{c_{1}}\right),\cdots \;\right),\\ & & \;U_{f}\left(\mu \right)=\left(\begin{array}{ccccc} 0 & & 0 & & 1\\ 1 & & 0 & & \tilde{\mu }\\ -1 & & 0 & & \tilde{\mu }\\ 0 & & 1 & & \tilde{\mu }\\ 0 & & -1 & & \tilde{\mu } \end{array}\right)\text{ with }\tilde{\mu }=\frac{\mu }{\sqrt{2}}. \end{array}$$
(26)

Assuming the contact force $f_{c}={\left(f_{x}^{c_{1}},f_{y}^{c_{1}},f_{z}^{c_{1}},\cdots \;\right)}^{⊤}$ lying in the friction cone, we would want to find **f**_*c*_ that is located as close to the center of the cone as possible as shown in Figure 9. In this manner, we can formulate the problem:

$$\begin{array}{ccc} & \min & \underset{c\in C}{\sum }\frac{1}{2{\left(\mu^{c}\right)}^{2}}\left({\left(f_{x}^{c}-{\tilde{\mu }}^{c}f_{z}^{c}\right)}^{2}+{\left(f_{x}^{c}+{\tilde{\mu }}^{c}f_{z}^{c}\right)}^{2}+{\left(f_{y}^{c}-{\tilde{\mu }}^{c}f_{z}^{c}\right)}^{2}+{\left(f_{y}^{c}+{\tilde{\mu }}^{c}f_{z}^{c}\right)}^{2}\right)\\ & & =\underset{c\in C}{\sum }\left(\frac{{\left(f_{x}^{c}\right)}^{2}+{\left(f_{y}^{c}\right)}^{2}+2{\left({\tilde{\mu }}^{c}f_{z}^{c}\right)}^{2}}{{\left(\mu^{c}\right)}^{2}}\right)≜f_{c}Wf_{c}^{⊤},\;\text{where }W=\mathrm{diag}\left(\frac{1}{{\left(\mu^{c_{1}}\right)}^{2}},\frac{1}{{\left(\mu^{c_{1}}\right)}^{2}},1,\cdots \;\right),\\ & \text{s.t. } & Af_{c}=b. \end{array}$$

Note that we used weighted sum to prioritize the slippery contact. Then solving constrained minimization problem, we get $f_{c}=W^{-1}A^{⊤}{\left({AW}^{-1}A^{⊤}\right)}^{-1}b$ .

### 8.3 Proof of proposition 1

Given the bounded function *f*_min_ ≤ *f*(*t*) ≤ *f*_max_, *∀t* ∈ (0, *T*) and *∃s* ∈ (0, 1) s.t. *f*(*t*) = *sf*_min_ + (1 − *s*)*f*_max_, *∀t* ∈ (0, *T*). Then *s*, (1 − *s*) ≥ 0 and

$$\begin{array}{cc} \left(f\left(t\right)B_{1}+B_{2}\right)x+c & =\left(\left(sf_{\text{min}}+\left(1-s\right)f_{\text{max}}\right)B_{1}+B_{2}\right)x+c,\\ & =s\left(\left(f_{\text{min}}B_{1}+B_{2}\right)x+c\right)+\left(1-s\right)\left(\left(f_{\text{max}}B_{1}+B_{2}\right)x+c\right),\\ & \geq 0. \end{array}$$
